# Gleanings

**Published:** 1876-04

**Authors:** 


### GLEANINGS.

NORMAL OVARIOTOMY.—At the last meeting of the Medical Society of Virginia, Dr. J. Marion Sims, of New York, was invited to address the Society. "Also, in cases of prolapse of either ovary, Dr. Sims advises that pessaries should be let alone. In fact, in this latter class of cases—prolapse of the ovaries—he is disposed to the belief that the operation of *normal ovariotomy*, as proposed by Dr. Robert Battey, of Georgia, promises better results than anything else. This operation consists essentially in the removal of the normal ovary that has, however, become prolapsed. He has done this operation once, and he has on hand another case upon whom he proposes to perform the same operation next Monday, in New York. The lady upon whom he operated has gone as a missionary to China; her husband was formerly a practicing physician in one of our Western cities. She was the subject of both profuse menorrhagia and metrorrhagia, and had an irritable prolapsed ovary, for which he proposed this operation suggested by Dr. Battey—after having first fully acquainted her with the dangers to life of the operation. In performing it, the two leading objects he had in view were (1) to get rid of the irritable ovary, and (2) to perform the operation so as to let the anterior uterine ligaments hold up the uterus in its natural position. The operation was performed last February, and the patient was sufficiently well in a month to allow her to return to her home in the West. About three weeks ago, however, Dr. Sims received a letter from Dr. Scott, of San Francisco, stating that he had just seen the lady on her second return from China, and that she was then perfectly well."

Dr. John Barlett, President of the Chicago Society of Physicians and Surgeons, suggests an artificial placental respiration to maintain the life of the child in cases where the placenta is expelled long prior to the birth of the child—"as a means of saving some of the children born under these unfavarable circumstances," I suggest the following practice: "Directly upon the detachment of the placenta, or as soon thereafter as practicable, immerse it in the fresh, warm, defibrinated blood of some animal, as the sheep. Change the blood as often as necessary and practicable, and aerate it and remove from it the accumulating carbonic acid by passing through it a stream of oxygen gas."

Dr. E. C. Barrett reports to the Virginia State Medical Society the case of a young lady of seventeen, who had always menstruated regularly from the rectum and never from the uterus. Her health was excellent, her physical development perfect. She married, became pregnant and the rectal menses ceased; she was normally delivered in due time and nursed her child. The rectal menses returned in fifteen months and continued regularly until the next pregnancy. She has now borne three children and there has been no menstruation at any time from the uterus, but always from the rectum. The general health has been wholly unimpaired.

The *Nashville Journal*, in a late editorial, grows jolly over the contemplation of *lager* as the future national beverage of America: "The effects of beer upon the organism are, in most instances, happy. Rarely is there a sufficient quantity absorbed to produce intoxication. In half an hour after its introduction into the stomach it has passed through the kidneys into the bladder, and serves thus as an excellent menstruem for the excretion of effete matters from the organism. There is nothing which so perfectly washes out the system as beer—nothing which leaves behind it so little unassimilable material. That day will be a happy one for America when beer shall have become the substitute for every other beverage, and when children are taught to take it with their mother's milk. Besides the direct physiological effects which it works, there are higher social feelings which follow its universal use, of which our country knows but little. Who can picture the contentment and good cheer of the German. *Beer Halle!*" etc.

In a late clinical lecture Prof. D. W. Yandell, of Louisville, Ky., treats the subject of erysipelas. He entertains the decided opinion that no local application is in any sense curative in this disease, nor does he believe that any constitutional remedy exercises a direct control over it. There are certain local applications which are soothing and comforting to the patient, and, therefore, beneficial, e. g., warm or cold fomentations, soothing powders or ointments. He enjoins watchful care for undue tumefaction or suppuration that timely incisions may be made to relieve the parts and prevent gangrene. In a general way, begin with an emetic, next a mercurial purge, then cooling diaphoretics. Forestall exhaustion by nourishing diet, brandy and tonics when needed. For delirium the potassic bromide. There is nothing specific in the action upon erysipelas of the muriated tincture iron, of quinine, the sulphites or potassic iodide. Iron may be useful as a tonic; the muriated tincture, especially so *when the urine* is albuminous. Quinine is useful (1) when a malarial influence is present, and (2) where the septic is in the ascendency.

Dr. Caro, of New York, expelled a tape worm, entire and fifty-three inches long, from a man who was taking half-ounce doses of copaiba every four hours.

"Dr. Briddon had never failed in the treatment of tape worm by the use of the ethereal extract of malefern. His usual plan was to administer the black draught, followed for twenty-four hours with nothing but beef teas; then from a dram to two drams of ethereal extract of malefern, and twelve hours afterwards a dose of castor oil."

Assistant Surgeon Buchanan, U. S. A., reports the case of an aged Mexican women who, while sleeping, received a deposit of ova from a fly which entered her nose. In great and protracted suffering there escaped from her nose, partly by the nares, partly by ulcerated openings in the soft palate and alæ nasi, no less than three hundred and twenty-five large magots. The woman recovered.

Dr. Sandridge, of Burksville, Ky., reports a case of large fibrous polypus of the nose successfully removed by traction upon the growth itself until its base of attachment, which was quite extensive, gave way and the entire growth was drawn out of the nostril.

Dr. Dora, of Illinois, reports a case in which the bladder was emptied by the aspirator twenty-seven times in the space of ten days.

Prof. Crowe, of Louisville, reports very favorably his experience in the use of the new Californian remedy, *grindelia robusta*, in asthma and dyspnœa.

Dr. Woollen, of Indiana, commends from his observation Squibb's fluid extract of leptandra as a mild and efficient purgative. He couches its unpleasant taste with some flavored syrup.

Dr. Maury, of Kentucky, reports a case in which he extracted a piece of brushwood twelve inches in length and one inch in diameter from the rectum of a farmer. Patient did well and returned to his work in ten days.

Dr. Eugene Smith reports, in the *Detroit Review*, a case of resuscitation from apparent chloroform death by the method of Nelaton—i. e., inverting the body that the blood may gravitate to the brain. The little patient lost both respiration and circulation three times, and was three times resuscitated by the method.

Dr. Dox, of Geneva, N. Y., gives the following description of his management of a *shoulder presentation* by postural treatment: "I folded several quilts compactly, laying them upon each other to the height of about one foot, and assisted her to kneel upon the quilts, with her head and shoulders resting upon the bed, and her face forward, so as to bring her body to an angle with the bed of nearly ninety degrees. I then pressed my hand gently against the shoulder, which readily receded, until I was enabled to clasp the vertex with my fingers, and with the assistance of the next pain to so engage it that, when the patient was placed upon her left side and the quilts removed, a perfectly natural presentation presented itself. In a few hours the labor terminated in the delivery of a healthy boy, weighing ten pounds. Only a few moments were occupied in the process, and subsequent experience convinces me that shoulder presentations can generally be converted in this way into natural ones, without a resort to turning, and with no risk to the mother or the child."

---

